# Enhancing implementation and compliance of the Screening Instrument for Child Abuse and Neglect (SCAN) in emergency departments in the Netherlands

**DOI:** 10.1136/bmjpo-2025-003362

**Published:** 2026-02-24

**Authors:** Eline A L van den Heuvel, Erica de Vries, Brita M de Jong-van Kempen, Roel Bakx, Renske F Bos, Pepijn van Empelen, Feline Hoedeman, Teus H Kappen, Ingrid M B Russel-Kampschoer, Patrycja Puiman, Maartje C M Schouten, Rian Teeuw, Cecile J Zwaans, Sanne L Nijhof, Elise M van de Putte

**Affiliations:** 1 Wilhemina Children’s Hospital - Social Paediatrics, University Medical Centre Utrecht, Utrecht, The Netherlands; 2 Paediatrics, Spaarne Hospital, Haarlem, The Netherlands; 3 Paediatrics, Medical Centre Leeuwarden, Leeuwarden, The Netherlands; 4 Paediatric Surgery - Paediatric Surgical Centre, Amsterdam University Medical Centres, Amsterdam, The Netherlands; 5 Paediatrics, Elkerliek Hospital, Helmond, The Netherlands; 6 Child Health, Netherlands Organization for Applied Scientific Research, Leiden, The Netherlands; 7 Sophia Children’s Hospital - General Paediatrics, Erasmus MC University Medical Center Rotterdam, Rotterdam, The Netherlands; 8 Wilhemina Children’s Hospital - Anaesthesiology, University Medical Centre Utrecht, Utrecht, The Netherlands; 9 Information Technology, University Medical Centre Utrecht, Utrecht, The Netherlands; 10 Paediatrics, University Medical Centre Groningen Beatrix Childrens Hospital, Groningen, The Netherlands; 11 Emma Children’s Hospital - Social Paediatrics, Amsterdam UMC Locatie AMC, Amsterdam, The Netherlands; 12 Paediatics, Jeroen Bosch Hospital, ’s Hertogenbosch, The Netherlands

**Keywords:** Child Abuse, Data Collection, Health Policy, Child Health

## Abstract

**Study purpose:**

This study examines the implementation of the Screening Instrument for Child Abuse and Neglect (SCAN) and the Structured Tool for Evaluating Positive Screened cases (STEPS) in nine emergency departments (EDs). The study aimed to assess whether implementation could enhance compliance with SCAN and to evaluate the usability of SCAN&STEPS within an implementation-focused design, in response to the decline in child maltreatment recognition observed in Dutch EDs over the past decade.

**Methods:**

SCAN&STEPS was embedded in ED workflow and electronic health records (EHRs), supported by a selection of implementation strategies, including e-learning, policy manuals and an awareness campaign. Effectiveness was assessed through compliance with SCAN, comparing preimplementation and postimplementation screening rates, with a ≥10% increase defined as clinically relevant. Usability of SCAN&STEPS was examined using a mixed-method design combining the Measurement Instrument for Determinants of Innovations and semistructured interviews. Subgroup analyses were conducted by hospital type, EHR, profession and years of working experience.

**Results:**

After implementation, the average compliance rate increased from 57.5% to 70.5%, with 3 of 8 sites achieving ≥10% improvement. Rates varied by site, with university hospital EDs having the highest improvement. Compliance was influenced by EHR configurations. Usability analysis identified five facilitators (perception of responsibility, social support, self-efficacy, knowledge and formal management ratification) and one barrier (unsettled organisation). Users considered SCAN&STEPS user-friendly, though perceived support differed between nurses and physicians due to role-specific factors.

**Relevance:**

SCAN&STEPS can improve compliance in recognising child maltreatment concerns, but tailored strategies are needed for further implementation in Dutch hospitals. The standardised approach enhances uniform data collection, enabling comparative analysis and interdisciplinary collaboration, advancing early detection of child maltreatment. Broader international adoption should account for policy and system-level differences and requires further validation to ensure applicability beyond the Dutch context.

**WHAT IS ALREADY KNOWN ON THIS TOPIC**

- Child maltreatment is a pervasive global issue with serious consequences. Emergency departments (EDs) play a vital role in early recognition. Screening instruments are intended to support early identification, yet their implementation and effectiveness remain debated, particularly regarding universal screening versus targeted case-finding approaches. In Dutch EDs, recognition rates had declined over the past decade despite mandated screening policies.

**WHAT THIS STUDY ADDS**

- Implementation of the Screening instrument for Child Abuse and Neglect (SCAN), supported by the Structured Tool for Evaluating Positive Screened follow-up, improved compliance with mandated universal screening at EDs. This combined approach supports broad awareness through screening while reserving comprehensive assessment for children with identified concerns and was considered usable by clinicians. Usability analysis identified multiple facilitators and one organisational barrier, with role-specific differences between nurses and physicians.

**HOW THIS STUDY MIGHT AFFECT RESEARCH, PRACTICE OR POLICY**

- SCAN’s standardised approach may support proportional and consistent early recognition of child maltreatment and facilitate comparative research and interdisciplinary collaboration. Broader international implementation requires tailored strategies, optimised electronic health record configurations and attention to system-level differences between countries.

## Introduction

 Child maltreatment is a pervasive global issue with far-reaching consequences, affecting millions of children each year.^1^ The profound impact is evident in the heightened morbidity and mortality rates among victimised children.^2^ In addition to the personal harm, societal costs are substantial, with annual economic losses for adverse childhood events (including maltreatment) in the Netherlands estimated at US$28.1 billion, representing 3.1% of the country’s gross domestic product.^3^

Early recognition of child maltreatment is crucial to prevent further harm and reduce long-term consequences.^4^,^6^ Screening instruments support this by providing clinicians with a structured framework for assessment and referral decisions, yet only 29% of the European emergency departments (EDs) use a screening instrument such as ESCAPE and SPUTOVAMO.^7^,^10^ These instruments have been shown to improve detection rates for concerns of maltreatment, but results about the detection of actual cases of maltreatment are varying depending on the chosen outcome parameter and setting.^7^,^14^ Barriers to recognising possible maltreatment in the ED include the absence of validated screening instruments, comprehensive hospital policies and the lack of staff training.^7 15 16^

**Figure 1:**
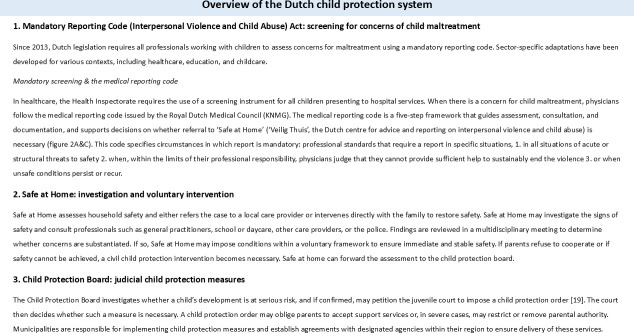
Dutch child protection system.

Over the past decade, the estimated prevalence of child maltreatment in the Netherlands remained unchanged.^17 18^ In cases with concerns for child maltreatment, Dutch physicians do not have a statutory duty to report, but they are required to follow the mandatory reporting code. This code provides a structured framework for deciding whether concerns should be referred to ‘Safe at Home*’,* the national centre for advice and reporting on concerns for child abuse and interpersonal violence (figure 1).^19^,^21^

**Figure 2:**
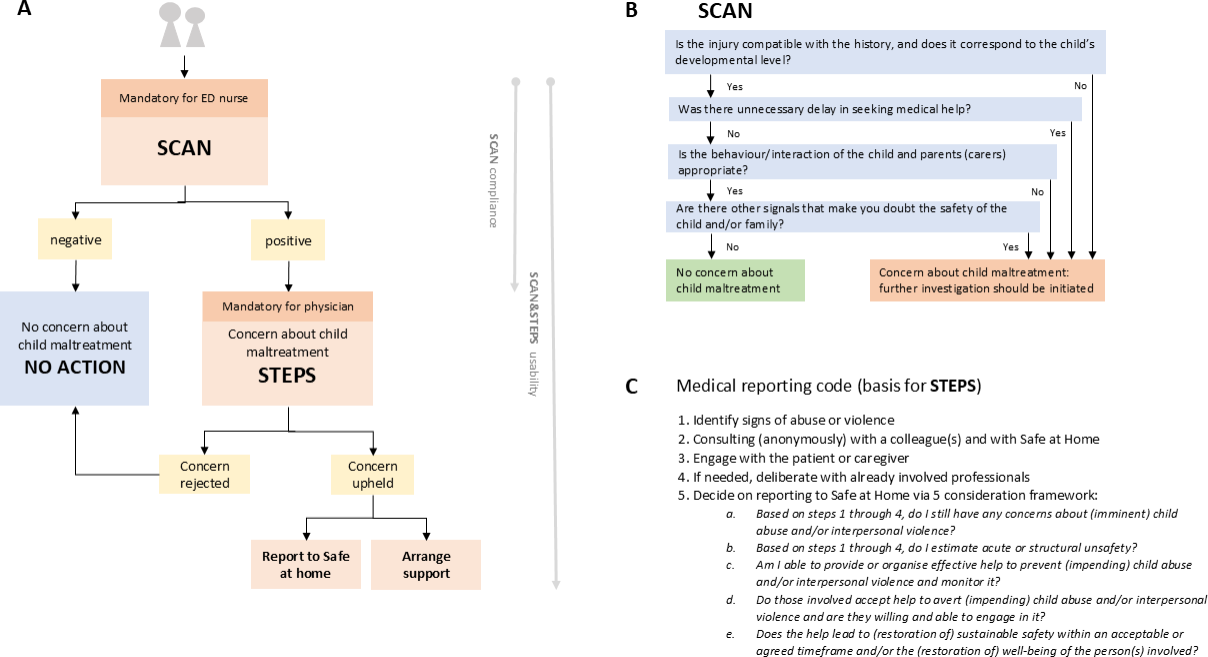
(**A**) SCAN&STEPS work process within the EHR, mandatory actions in bold; work process (arrows) with specific study components. (**B**) SCAN.^25^ (**C**) Medical reporting code, basis for STEP.^21^ ED, emergency department; EHR, electronic health record; SCAN, Screening instrument for Child Abuse and Neglect; STEPS, Structured Tool for Evaluating Positive Screened.

Although the prevalence of child abuse remained stable, reports to Safe at Home by hospital-based healthcare professionals have declined.^22 23^ This suggests reduced recognition of possible maltreatment, despite mandatory staff training on child abuse recognition mandated by the Inspectorate of Health.^24^ A possible contributing factor may be the lack of a uniform, feasible and validated screening instrument, as existing tools (eg, ESCAPE, SPUTOVAMO) showed variability in usability, content and validity. This resulted in variable maltreatment recognition protocols and inconsistent hospital policies and performance. These inconsistencies hinder interhospital comparability, limiting opportunities for shared learning and system-wide healthcare improvement. To address this hiatus, the Screening instrument for Child Abuse and Neglect (SCAN) (figure 2B) was developed as a brief, uniform and validated screening instrument. SCAN was derived from the data from three large validation studies of SPUTOVAMO, SPUTOVAMO-R and ESCAPE (after harmonisation of questions and outcome) and uses the most discriminative questions to improve validity and to ensure maximal feasibility and adherence in daily ED practice.^25^

This study aims to evaluate whether the implementation of SCAN in the ED of nine Dutch hospitals can improve compliance and thereby strengthen the recognition of child maltreatment concerns, within an implementation-focused evaluation. To support subsequent medical diagnostic follow-up and guidance in any safety concerns, the Structured Tool for Evaluating Positive Screened cases (STEPS) provides physicians with standardised guidance for navigating the medical reporting code. The second aim for this study is to evaluate the usability of the SCAN&STEPS in daily practice. The results are intended to support the nationwide implementation of the SCAN&STEPS, with the overall goal of improving the early identification of child maltreatment.

## Methods

### Study design

An observational mixed-methods study was conducted. Compliance with SCAN was assessed quantitatively by comparing the compliance rate, defined as the proportion of eligible children in the ED who were screened using a screening instrument, preimplementation and postimplementation. A clinically relevant effect was defined as an increase of ≥10% of the compliance rate.

The usability of the SCAN&STEPS was evaluated through a combination of data sources. This included quantitative data from the Measurement Instrument for Determinants of Innovations (MIDI) and insights gathered from in-depth semistructured interviews. Descriptive characteristics of MIDI respondents were obtained.

To ensure broad applicability of the SCAN&STEPS, a purposeful sample of hospitals was selected, including three university hospitals and six non-university hospitals. These 9 hospitals, collectively operating 12 ED locations, are all hospital-based and provided care for patients under 18 years (among other age groups).

### Patient and public involvement

Patients or the public were not involved in the design, conduct, reporting or dissemination plans of this research.

### Preparatory to SCAN implementation

Three preparatory steps to effectively implement SCAN&STEPS were designed to address known barriers and facilitators.^15 16^ First, the STEPS was developed to support physicians in clinical decision making for SCAN-positive cases, providing a systematic approach to assessing concerns about child maltreatment. The tool offers clarifying questions per step of the medical reporting code (available on request), thereby integrating medical follow-up with the mandated procedures: the diagnostic phase (forensic medical consultation, peer review, additional examinations of the child, and advice from Safe at Home; figure 2C, steps 1–2), the communication phase (dialogue with parents, child and involved care providers; figure 2C, steps 3–4), and the treatment phase (safeguarding measures based on the final assessment, ranging from follow-up within healthcare services to referral for further interventions in the child protection chain, such as Safe at Home; figure 2C, step 5). Second, SCAN&STEPS was integrated into the ED work process and incorporated into two widely used electronic health record (EHR) systems. Within this work process (figure 2A), agreements were established, assigning nurses the responsibility of the SCAN completion. When SCAN indicates a concern for child maltreatment, by one or more deviant answers (SCAN-positive), further evaluation is necessary and physicians are supported in navigating the medical reporting code by STEPS. To ensure seamless follow-up, a screen-positive outcome is directly linked to the STEPS within the EHR. All children (<18 years) presented at the ED were eligible for the use of SCAN&STEPS; there were no exclusion criteria. Third, to support the implementation, several resources were developed: (1) a concise e-learning to educate professionals on the SCAN&STEPS work process; (2) a policy manual outlining the process including the SCAN&STEPS, communication strategies and legal guidelines (available on request) and (3) awareness campaign materials to prepare the staff for the SCAN&STEPS implementation. All resources were provided to participating hospitals at no cost.

### Implementation strategy

Before implementation, hospitals were required to fulfil specific inclusion criteria: (1) appoint a clinical ambassador, (2) integrate the SCAN&STEPS into the EHR system and (3) provide consent for data collection.

The clinical ambassador determined the start date for implementation. In collaboration with the research team, the SCAN&STEPS implementation strategy was customised. The clinical ambassador received tailored guidance on critical decisions, including configuring the EHR system (workflow interruptions and system notifications), selecting colleagues for mandatory training delivered through the e-learning (at minimum: ED staff and paediatricians), and allocating resources for awareness campaigns. The research team provided continuous support throughout the implementation process to ensure uniform and effective execution. A more detailed description of the implementation strategy can be found in online supplemental file A.

### Data collection

To assess the compliance with the use of a screening instrument, data collection began 3 months before implementation and continued for at least 1 year. Aggregated data were collected from each participating hospital, including the total number of ED visits by children under 18 years, the number of children screened (using any instrument), and the number of screened-positives.

To evaluate the usability of the SCAN&STEPS, a multiphase, mixed-method approach was used. First, users of SCAN&STEPS identified factors influencing implementation in their hospital. At each site, the clinical ambassador purposively selected a broad group of SCAN&STEPS users (a minimum of 30–50, depending on the hospital size), who received the MIDI questionnaire via email at least 3 months postimplementation; up to two reminders were sent. Respondents answered anonymously and were asked to provide background information, including current profession, number of years of work experience and experience with child abuse screening instruments and reporting to Safe at Home.

Subsequently, the clinical ambassador at each hospital purposively selected a smaller, representative sample of SCAN&STEPS users, including at least a paediatrician, an ED physician and an ED nurse with whom semistructured in-depth interviews were conducted. An interview guide was developed by the research team to ensure consistency (see online supplemental file B). These interviews, conducted in hybrid format via Microsoft Teams and at least 2 months after distribution of the MIDI questionnaire, followed guided questions informed by the hospital-specific MIDI results and explored facilitators and barriers in greater detail. Findings were analysed using a descriptive, deductive content summarisation approach, in which responses were categorised as facilitators or barriers and summarised at the centre level. Draft summaries were reviewed within the research team to ensure consistency. A formal thematic analysis was not conducted, as the study aimed to capture centre-specific facilitators and barriers for structured comparison across sites rather than to develop new themes or theoretical constructs.

SCAN&STEPS implementation took place sequentially between May 2021 and March 2022.

### MIDI questionnaire

The questionnaire used was adapted from th MIDI,^26^ an evidence-based framework that outlines 29 determinants influencing healthcare innovation implementation. These determinants were organised into four domains: (1) the innovation, (2) the user, (3) the organisation and (4) the sociopolitical context.

From the original 29 MIDI determinants, the research team identified 17 as potentially relevant (of which 3 included a subquestion). These determinants were adapted to reflect the context of the SCAN&STEPS implementation, in accordance with instructions of the MIDI questionnaire use. The questionnaire consisted of items rated either on a 5-point Likert scale (n=18), ranging from ‘totally disagree’ (1) to ‘totally agree’ (5) or binary response options (n=2) (table 1).

**Table 1:** MIDI determinants adjusted for SCAN&STEPS implementation

Level of innovation determinants	Specific determinant	Adapted MIDI question
Determinants associated with the innovation (SCAN&STEPS)	1. Procedural clarity	SCAN&STEPS clearly specifies the activities I need to perform and their required sequence.
	2. Completeness	SCAN&STEPS provides all the necessary information and materials for effective use.
	3. Complexity*	SCAN&STEPS is too complicated for me to use.
	4. Compatibility	SCAN&STEPS aligns well with how I am used to work.
	5. Relevance to patient	I consider SCAN&STEPS appropriate for my patients.
Determinants associated with the adopting user (healthcare professionals)	6. Personal advantages and disadvantages	To what extent does using the SCAN&STEPS provide personal advantages or disadvantages? Using SCAN&STEPS helps me spend less time determining the necessary actions when following the medical reporting code. Using SCAN&STEPS gives me greater confidence that I am documenting in compliance with legal requirements.
	7. Outcome expectations	I consider it important to better recognise signs of child abuse in my patients through the SCAN. I expect the SCAN to help me effectively identify signs of child abuse in my patient.
	8. Perception of responsibility	I consider it part of my professional duties to use the SCAN&STEPS.
	9. Social support	I can rely on sufficient support from my colleagues if I require assistance in using the SCAN&STEPS.
	10. Self-efficacy	To what extent do you believe you can successfully complete the following steps? Completing the SCAN. Completing the STEPS.
	11. Knowledge	I have sufficient knowledge to use SCAN&STEPS.
Determinants associated with the organisation	12. Formal ratification by management†	Has your organisation formally established agreements, through management or professional bodies, regarding the use of the SCAN&STEPS?
	13. Staff capacity	There is sufficient staffing in our organisation to use the SCAN&STEPS as intended.
	14. Availability of materials and resources	My organisation provides me with adequate materials and resources to use the SCAN&STEPS as intended.
	15. Unsettled organisation†*	Apart from the implementation of the SCAN&STEPS, are there other ongoing or upcoming changes in your organisation (eg, restructuring, mergers, budget cuts, staff turnover, or other innovations)?
	16. Access to information on SCAN&STEPS use	I can easily access information about the use of SCAN&STEPS within my organisation.
Determinants associated with the sociopolitical context	17. Legislations and regulations	The activities outlined in SCAN&STEPS align with the medical reporting code on child abuse and interpersonal violence.

* Reverse scale.

† Binary question.

MIDI, Measurement Instrument for Determinants of Innovations; SCAN, Screening Instrument for Child Abuse and Neglect; STEPS, Structured Tool for Evaluating Positive Screened.

To ensure the appropriateness of the selected determinants, a preliminary in-depth interview was conducted, 2 months after the SCAN&STEPS implementation in the first hospital but before distributing the MIDI-questionnaire. This interview involved a representative sample of users and aimed to identify facilitators and barriers to SCAN&STEPS implementation. The findings closely aligned with the selected MIDI determinants, affirming their relevance. Consequently, no further modifications to the MIDI determinants were made. The MIDI questionnaire was hereafter analysed prior to conducting the semistructured interviews.

### Data analysis

IBM SPSS Statistics for Windows (V.29.0.1.0, IBM) was used for quantitative data analysis. Descriptive statistics were applied to assess the compliance to screening and usability of SCAN&STEPS from the MIDI questionnaire (including mean, IQR, percentages, SD and χ² tests).

Consistent with prior research, MIDI determinants phrased positively were classified as facilitators if ≥80% of participants responded with ‘agree’ or ‘strongly agree’.^27^ Conversely, determinants were categorised as barriers if ≥20% of participants responded with ‘disagree’ or ‘totally disagree’. The reliability of the SCAN&STEPS-adapted MIDI-questionnaire was evaluated using Cronbach’s alpha, which indicated good internal consistency (α=0.85).

Prespecified subgroup analyses examined differences in compliance rate and usability by hospital and EHR type. Subgroup analysis for usability was furthermore performed by work experience (early career (<5 years), mid-career (6–15 years), experienced (>15 years)) and type of profession (nurse, physician) to account for task-specific responsibilities within the SCAN&STEPS workflow. In accordance with standard practice, a p<0.05 was used to define statistical significance in subgroup analysis.

## Results

All participating hospitals fulfilled the inclusion criteria and were able to implement the SCAN&STEPS within the study period.

### Compliance with SCAN screening

Prior to the implementation, all hospitals employed a child maltreatment screening instrument, with an average compliance rate of 57.5% (range: 11%–100%). Postimplementation, the compliance increased to 70.8% (range: 24%–100%) (table 2). The proportion of children visiting the ED who screened positive remained consistent, at 2.5% preimplementation (range: 0.9%–4.0%) and 2.3% postimplementation (range: 0.7%–6.5%).

**Table 2:** ED presentation of children preimplementation (left) and postimplementation

Hospital	Preimplementation							Postimplementation					
	Preimplementation tool	Children at ED	Screened children	Screened children	Screen positive	Screen positive/ children at ED	Period	Children at ED	Screened children	Screened children	Screen positive	Screen positive/ children at ED	Period
		Total (n)	Total (n)	%	Total (n)	%	Months	Total (n)	Total (n)	%	Total (n)	%	Months
A*	SL	618	618	100	25	4.0	3	6564	5945	90.6	211	3.2	23
B†	ES	1115	131	11.8	33	2.9	3	5953	3154	53	210	3.5	20
C†	SR	811	799	98.5	35	4.3	3	3834	3834	100	250	6.5	16
D*	S	1115	597	53.5	17	1.5	3	7459	5059	67.8	118	1.5	15
E*	SL	1663	497	29.9	15	0.9	3	14 443	3559	24.6	170	1.2	13
F†	S	1565	402	25.7	26	1.7	3	15 621	11 008	70.5	244	1.5	21
G*	S	2091	1376	65.8	50	2.3	3	22 199	14 073	63.4	247	1.1	22
H*	S	772	576	74.6	21	2.7	3	6014	4258	70.8	41	0.7	14
I*	S							2266	2121	93.6	26	1.5	12
Total		9750	4996		222		24	84 353	53 011		1517		156
Average				57.5		2.5	3			70.5		2.3	17

Hospital I was not able to retrieve the preimplementation data because the implementation started simultaneously with a go-live of a new EHR.

EHR type: A–D = Chipsoft; E–I = EPIC.

* Non-university hospital.

† University hospital.

ED, emergency department; EHR, electronic health record; ES, ESCAPE; S, SPUTOVAMO; SL, SPUTOVAMO-like (locally adjusted SPUTOVAMO); SR, SPUTOVAMO-R.

Compliance rates increased in four of the eight hospitals, with three achieving gains exceeding >10%. In the other hospitals, compliance rates decreased (range 2.4%–9.4%). At hospital A, a reorganisation of emergency care services during the COVID-19 pandemic led to the erroneous inclusion of children assessed outside the ED as ED cases in the postimplementation data. This misclassification contributed to a 9.4% compliance rate decrease. Despite this, hospital A maintained a higher compliance rate than most EDs due to its strong preimplementation performance.

Subgroup analysis showed that there were significant differences in compliance rates between university and non-university hospital EDs preimplementation (45% vs 65%, p<0.001). After implementation, the difference reversed (75% vs 68%, p=0.30) with university EDs outperforming non-university EDs.

Differences in compliance rate were also observed between different EHR systems. Three of four Chipsoft hospitals increased compliance rates, compared with one of four EPIC hospitals, though this was not statistically significant (p=0.1). Hospitals A–D implemented a ‘stop function’ in the EHR system requiring ED nurses to complete the SCAN before finalising the consultations. Variations in default settings within Chipsoft affected compliance levels, with hospital C achieving 100% compliance under optimised conditions. In contrast, hospitals E–I lacked this functionality in their EHR system.

During the in-depth interviews, participants reported that despite the potential ‘stop function’ hospitals hesitated to implement it due to concerns about delaying the transfer of critically ill patients from the ED to the intensive care unit or operating room if the SCAN was incomplete. Other barriers expressed were workload pressure, leading to the SCAN being frequently overlooked or omitted. Hospital E attributed low compliance to high staff turnover and infrequent training on recognising abuse, limiting the integration of SCAN into routine practice for many professionals.

SCAN users acknowledged the benefits of SCAN&STEPS, particularly due to improved workflow agreements. One nurse working in hospital G explained, “*Although the compliance rate did not increase, the workflow in the emergency department changed significantly. Prior to implementation, the SPUTOVAMO was often completed retrospectively by members of the child abuse team, mostly after the child had already left the emergency department. With the SCAN&STEPS workflow agreements, this responsibility lies explicitly with the treating nurse who actually sees the patient. This is more accurate and also enabled the child abuse team to focus more on complex cases rather than on completing an instrument*.”

### Usability of SCAN&STEPS

The MIDI questionnaires were administered on average 5 months after the implementation (range: 2–9 months). A response rate could not be determined due to the distribution method, as local ambassadors distributed the questionnaire link via email to (groups of) SCAN&STEPS users or included it in departmental newsletters. Of the 259 healthcare professionals who participated in the questionnaire, 173 completed the questionnaire in its entirety. Characteristics of MIDI responders are shown in table 3.

**Table 3:** Characteristics of healthcare providers participating in MIDI questionnaire in nine hospitals

	Physician (n)	Nurse (n)	Other (n)	Total (n, %)
Hospital type				
University (n=3)	43	32	7	82 (32)
Non-university (n=6)	41	113	18	172 (68)
Total	84	145	25	254 (100)
Work experience				
Years in current job (mean, SD)	10 (±7.3)	14 (±10.3)	11 (±9.3)	
Experience with a child abuse screening instrument	84	143	23	250 (98.4%)
Experience with reporting to safe at home	84	141	23	248 (97.6%)

* Other healthcare professionals include physician assistants/nurse practitioners, medical secretaries and PhD students

MIDI, Measurement Instrument for Determinants of Innovations; PM, pro memorie.

In-depth interviews were conducted on average 2.5 months after the administration of the MIDI questionnaire (range: 1–5 months). A total of 46 professionals participated, with an average of five participants per hospital.

### Facilitators

MIDI questionnaires found five facilitators of impact when implementing the SCAN&STEPS (table 4).

**Table 4:** MIDI results of implementation facilitators and barriers of the SCAN&STEPS

MIDI item	Respondents (N)	Median (IQR)	Disagree/totally disagree (%)	Neutral (%)	Agree/totally agree (%)
1. Procedural clarity	213	4 (4–4)	5.6	16.9	77.5
2. Completeness	205	4 (3–4)	6.3	26.3	67.3
3. Complexity*	204	4 (3-4)	65.2	25.5	9.8
4. Compatibility	203	4 (3–4)	8.9	27.1	64
5. Relevance to patient	200	4 (4–4)	4.5	19	76.5
6a. Personal advantages/disadvantages—actions	188	3.5 (3–4)	13.8	36.2	50
6b. Personal advantages/disadvantages—documentation	186	3 (3–4)	9.7	41.9	48.4
7a. Outcome expectations—recognition	184	4 (3.25–4)	4.8	20.1	75
7b. Outcome expectations—expectance	181	3 (3–4)	9.4	55.2	35.4
8. Perception of responsibility	181	4 (4–5)	2.3	7.7	90.1
9. Social support	181	4 (4–4)	2.2	14.4	83.4
10a. Self-efficacy—SCAN	180	4 (4–4)	0.6	8.9	90.5
10b. Self-efficacy—STEPS	180	4 (3–4)	4.4	29.4	66.3
11. Knowledge	179	4 (4–4)	3.4	16.2	80.4
12. Formal ratification by management†	177	2 (2–2)	13		87
13. Staff capacity	175	4 (3–4)	6.3	28.6	65.1
14. Availablility of materials and resources	175	4 (3–4)	3.5	25.7	70.9
15. Unsettled organisation†*	175	2 (1–2)	44.6		55.4
16. Access to information on SCAN&STEPS use	175	4 (4–4)	2.3	20.6	77.1
17. Legislations and regulations	173	4 (4–4)	2.3	19.1	78.6

* Reverse scale.

† Binary question.

MIDI, Measurement Instrument for Determinants of Innovations; SCAN, Screening Instrument for Child Abuse and Neglect; STEPS, Structured Tool for Evaluating Positive Screened.

Four of these were in the domain of the user and one in the domain of the organisation. Participants provided the following descriptions during the interview:

#### Perception of responsibility

90% of respondents acknowledged that recognising child maltreatment is a fundamental aspect of their professional duties. They emphasised their pivotal role in identifying potential unsafe situations, noting that their focused yet brief interactions with children and parents at the ED provide critical opportunities for recognition. As one respondent stated, “*SCAN strengthens my ability as a nurse to effectively recognise signs of child abuse. It helps me articulate concerns, enabling clearer communication with the physician”*.

#### Social support

83% of respondents reported that they can rely on their colleagues for support when completing the SCAN&STEPS. Many respondents remark, “*In the emergency department, an experienced colleague is available almost 24/7, providing easy access for consultation when I have doubts about signs of child abuse*.”

#### Self-efficacy SCAN

Over 90% of respondents reported that they are able to successfully complete the SCAN, describing it as brief, clear and user-friendly. As one respondent noted: “*The SCAN is highly practical and represents an improvement over the SPUTVAMO*”.

#### Knowledge

80% of respondents say they have sufficient knowledge to work with SCAN&STEPS. They highlighted the availability of adequate resources, such as the manual and e-learning. However, during the interviews, variability in knowledge among professionals was identified as a significant challenge, potentially resulting in interprofessional discrepancies and missed signs of child maltreatment. Continuous training was repeatedly emphasised as critical to improve detection of possible signs of maltreatment, as illustrated by one respondent: “*When you are not on duty for a while, the information tends to fade, so maintaining continuous attention to training is essential.”*

#### Formal ratification by management

87% of respondents reported receiving support from their management, primarily through formal working agreements regarding the use of SCAN&STEPS. One respondent remarked: “*It’s very clear that the SCAN is mandatory and that it must be completed by default. Agreements have been made about this, and we are reminded of it verbally*.”

Respondents from hospitals without prior formal agreements highlighted the significant advantages of implementing default SCAN and workflow for all children within the EHR. They reported that this approach increased compliance rates by addressing inconsistent practices: previously, the screening instrument was completed by either the physicians or the nurses, and often not at all due to assumptions that another professional would handle it.

### Barrier

One key barrier identified was organisational instability (unsettled organisation). 44% of respondents reported that various organisational factors adversely affected the implementation of SCAN&STEPS. The most frequently cited was the COVID-19 pandemic, as SCAN&STEPS was introduced during the height of the crisis. Respondents emphasised that the high workload and uncertainty associated with the pandemic reduced their readiness for change and willingness to engage in learning activities.

Other organisational factors mentioned included staff shortages, high staff turnover with insufficient time for comprehensive onboarding, hospital or site mergers and reorganisations, and changing EHR system. One respondent summarised: “*Turbulence in the emergency department occasionally complicates the completion of the SCAN. This turbulence is not only related to workload but also to structural changes within the ED. These factors redirected time and attention away from the SCAN&STEPS*.”

### Subgroup analysis

#### Profession

Subgroup analysis of the MIDI questionnaire by profession identified three additional facilitators for nurses (all in the domain of the innovation), and two additional barriers for physicians (one in the user domain and the other in the organisation domain) (table 5).

**Table 5:** Subgroup analysis MIDI determinants—profession (left: physicians)

MIDI item	Physicians	Mean (SD)	Disagree/ totally disagree	Neutral	Agree/ totally agree	Nurses	Mean (SD)	Disagree/ totally disagree	Neutral	Agree/ totally agree	χ^2^
	N		%	%	%	N		%	%	%	
1. Procedural clarity	68	3.62 (0.79)	8.5	22	69.5	124	3.80 (0.64)	4.2	10.4	85.4	0.11
2. Completeness	65	3.45 (0.73)	11.9	32.2	55.9	120	3.76 (0.58)	3.1	16.7	80.2	0.01
3. Complexity*	65	3.40 (0.97)	50.9	30.5	18.6	119	3.90 (0.86)	74	17.7	8.3	0.01
4. Compatibility	64	3.36 (0.86)	15.3	30.5	54.2	119	3.67 (0.67)	6.3	20.8	73	0.05
5. Relevance for patient	64	3.59 (0.78)	10.2	23.7	66.1	116	3.85 (0.55)	2.1	13.5	84.4	0.10
6a. Personal advantages/disadvantages—actions	62	3.18 (0.90)	25.4	35.6	39	106	3.50 (0.78)	8.3	36.5	55.2	0.04
6b. Personal advantages/ disadvantages—documentation	62	3.42 (0.67)	8.5	42.4	49.2	105	3.36 (0.72)	11.5	41.7	46.9	0.94
7 a. Outcome expectations—recognition	61	3.62 (0.84)	8.5	25.4	66.1	104	3.88 (0.63)	2.1	19.8	78.2	0.14
7b. Outcome expectations—expectance	60	3.17 (0.64)	10.2	66.1	23.7	103	3.35 (0.71)	10.4	50	39.6	0.14
8. Perception of responsibility	60	4.17 (0.89)	3.4	15.3	81.4	103	4.28 (0.63)	2.1	3.1	94.8	0.02
9. Social support	60	3.90 (0.77)	3.4	23.7	72.8	103	4.14 (0.59)	1	8.3	90.7	0.02
10a. Self-efficacy—SCAN	60	3.90 (0.71)	1.7	18.6	79.7	102	4.24 (0.51)	0	3.1	96.9	<0.01
10b. Self-efficacy—STEPS	60	3.80 (0.78)	5.1	20.3	74.6	102	3.61 (0.69)	5.2	32.3	62.5	0.25
11. Knowledge	60	3.70 (0.79)	8.5	18.6	72.9	101	3.96 (0.56)	1	13.5	85.4	0.12
12. Formal ratification by management†	60	1.78 (0.42)	20.3		79.7	99	1.91 (0.29)	9.4		90.6	0.03
13. Staff capacity	59	3.34 (0.84)	15.3	39	45.8	98	3.79 (0.50)	1	20.8	78.1	<0.01
14. Availability of materials and resources	59	3.53 (0.70)	6.8	33.9	59.3	98	3.82 (0.52)	1	20.8	78.1	0.07
15. Unsettled organisation*†	59	1.46 (0.50)	45.8		54.2	98	1.60 (0.49)	61.5		38.5	0.07
16. Access to information on SCAN&STEPS use	59	3.73 (0.72)	3.4	27.1	69.5	98	3.85 (0.56)	2.1	18.8	79.2	0.44
17. Legislations and regulations	59	3.95 (0.75)	3.4	20.3	76.2	96	3.83 (0.57)	2.1	19.8	78.1	0.05

* Reverse scale.

† Binary question.

MIDI, Measurement Instrument for Determinants of Innovations; SCAN, Screening Instrument for Child Abuse and Neglect; STEPS, Structured Tool for Evaluating Positive Screened.

Perceptions of personal advantages and disadvantages (subquestion ‘actions’) differed between nurses and physicians: A nurse stated: “*Before the SCAN&STEPS, we had no support in navigating the reporting code. I still probably won’t do it independently, but now, when I have to, I know how*”. In contrast, a physician noted: “*I don’t feel more confident documenting in a legally correct manner. It remains a judgment call, as every case is different. You can never completely eliminate this uncertainty in the medical reporting code*”.

These differences are caused by distinct roles, responsibilities and exposure: nurses routinely engage with SCAN, while physicians interact less frequently with STEPS, which is only activated in screen positive cases. Physicians cited the extensive medical reporting code and time-consuming nature of familiarising themselves with the STEPS as barriers. However, when physicians gained experience with STEPS, they noted that it made them more intuitive and supportive compared with preimplementation practices. For example, by the standardised STEPS documentation, which facilitates locating the mandatory reporting steps within the EHR, enabling appropriate actions regardless of changes in the responsible physician during the process. A barrier of the STEPS was the increased administrative burden, challenging its integration into clinical workflows.

The determinant of completeness also highlights the differences in responsibilities within the workflow and is furthermore reflected in significant variations across self-efficacy and formal ratification by management.

#### Hospital type

Subgroup analysis across hospital types identified two significant differences (see online supplemental file C). First, professionals in non-university hospitals more often considered their department as sufficiently staffed to work with the SCAN&STEPS, although this was not deemed a facilitator, as agreement did not reach the predefined threshold (>80%). Second, professionals in university hospitals more frequently reported organisational changes affecting implementation. While the in-depth interviews did not provide concrete explanations, these findings may reflect that academic hospitals are typically more dynamic environments due to training responsibilities and research activities, which can lead to greater organisational changes and higher staff turnover. Combined with reported perceptions of fewer staff resources, this may ultimately pose challenges to consistent compliance.

#### Working experience

The subgroup analysis of working experience indicated no clinically significant differences between professionals with different levels of working experience (detailed in online supplemental file C).

## Discussion

SCAN&STEPS was successfully implemented in nine Dutch EDs, achieving clinically significant increased compliance in three of the eight hospitals. Overall usability was good, as indicated by the MIDI outcomes and interview findings, which identified mostly facilitators. These results suggest that SCAN&STEPS can promote standardised practice and support clinical decision-making regarding potential child maltreatment.

### Compliance

Although all hospitals intended to use SCAN for *all* children visiting the ED, this goal was only achieved in one hospital. Compliance increased most in university hospitals, largely due to low baseline rates, eliminating significant postimplementation differences between university and non-university settings (75% vs 68%). In non-university hospitals, compliance remained largely stable. Variations in EHR configurations appear to influence compliance, with 100% compliance achievable under optimised conditions. Future implementation should prioritise EHR optimisation and digital workflow in collaboration with clinicians, IT teams and hospital management to ensure better alignment with daily practice. Given the low baseline compliance rates prior to implementation, further consolidation of SCAN at the national level is warranted, while persistent variation between hospitals underscores the need for tailored implementation strategies.

### Usability and professional roles

Overall, SCAN&STEPS was considered user-friendly and supportive of interdisciplinary collaboration, likely due to the range of support tools offered before and during implementation. Key facilitators were identified in the user domain, whereas organisational turbulence in emergency care emerged as a barrier. Nurses reported greater benefits than physicians, reflecting differences in roles and exposure: SCAN is applied to all children, while STEP is only used in screened positives, limiting physicians’ familiarity. Strengthening physicians’ training and embedding STEP more firmly into clinical workflow may improve further uptake.

### Universal screening versus case-finding

The Dutch context combines mandated universal application of SCAN with targeted case evaluation via STEPS for SCAN-positive cases.^19^ Intended to reduce the likelihood of missed cases, this hybrid strategy enhances professional awareness and promotes consistency, while safeguarding proportionality by ensuring that further steps are taken only when concerns are identified. It supports physicians in initiating dialogue with parents and reporting to Safe at Home only when necessary to ensure a child’s safety. In this way, the universal use of SCAN&STEPS fosters awareness and clinical enquiry, while avoiding unnecessary burden for families, thereby addressing key concerns expressed in line with WHO recommendations.^28 29^ Our findings show that compliance improved, more children were screened, and the proportion of positives remained stable at 2.5%, suggesting that SCAN may increase awareness. Whether this translates into improved protection of children remains uncertain. Rigorous evaluation of the full care pathway (including medical follow-up, forensic consultation, voluntary support and referral to Safe at Home) is needed to determine the predictive value and real-world effectiveness of SCAN&STEPS. The uniform nationwide application of SCAN&STEPS in the Netherlands offers a unique opportunity to generate insights on performance and outcomes, particularly given the ongoing debate on the effectiveness of universal and targeted approaches.^30^,^32^

### Strengths and limitations

This study’s strengths include a representative sample of Dutch EDs, a postimplementation follow-up of at least 1 year and a significant number of MIDI respondents, enhancing the generalisability of the findings. The average compliance rate of 70.8% of children presenting at EDs aligns with prior studies,^30^,^32^ as does the percentage of screen-positive cases (1.8%–2.5%).^30^,^33^ However, the short preimplementation period and use of aggregated pre/post data limited our ability to analyse hospital-level trends and screen-positive proportions using interrupted time series analysis. No data on physician subspecialties were collected, which may limit interpretation of potential specialty-specific differences. Diagnostic accuracy could not be assessed in this study; future research is needed to evaluate SCAN&STEPS’ performance.

### Future directions

Although the use of a screening instrument to detect child abuse at EDs in the Netherlands is mandated by the Health Care Inspectorate, legislation alone does not ensure consistent practice; effective implementation, training and monitoring are crucial to translate this requirement into meaningful improvements in the recognition and management of child maltreatment. Increased compliance is only meaningful if SCAN&STEPS are completed with careful knowledge and consideration, emphasising the need for continuous training on the topic. The SCAN&STEP combined approach is particularly relevant because no screening tool can adequately detect the full spectrum of child maltreatment and the positive predictive value will inevitably remain modest. Although earlier studies demonstrated high negative predictive values, these were tested within their own datasets rather than in real-world ED settings.^25^ Future research must therefore examine whether the combined use of SCAN&STEPS prevents missed cases and achieves the intended balance between sensitivity and proportionality.

National implementation of SCAN&STEPS standardises documentation and enables hospitals to monitor performance in recognising concerns for child maltreatment. Such data allow for comprehensive trend analyses, offering valuable insights to optimise hospital policies. Recent research underscores the importance of data integration in improving these strategies.^33^,^35^ In the near future, SCAN&STEP should also facilitate seamless data linkage from hospitals with Safe at Home, in a way that enables independent evaluation of the entire care chain. This will further enable evaluation across this chain, an essential step towards reducing the impact of child maltreatment. While SCAN&STEPS demonstrates clear potential within the Dutch context, broader international adoption should carefully account for variations in child abuse policies and healthcare systems.

## Supplementary material

10.1136/bmjpo-2025-003362 online supplemental file 1

10.1136/bmjpo-2025-003362 online supplemental file 2

10.1136/bmjpo-2025-003362 online supplemental file 3

## Data Availability

Data are available on reasonable request. All data relevant to the study are included in the article or uploaded as supplementary information.

## References

[1] World Health Organization (2020). Global status report on preventing violence against children.

[2] Gilbert R, Widom CS, Browne K (2009). Burden and consequences of child maltreatment in high-income countries. Lancet.

[3] Hughes K, Ford K, Bellis MA (2021). Health and financial costs of adverse childhood experiences in 28 European countries: a systematic review and meta-analysis. Lancet Public Health.

[4] Purewal Boparai SK, Au V, Koita K (2018). Ameliorating the biological impacts of childhood adversity: A review of intervention programs. Child Abuse Negl.

[5] Jenny C (1999). Analysis of Missed Cases of Abusive Head Trauma. JAMA.

[6] Deans KJ, Thackeray J, Askegard-Giesmann JR (2013). Mortality increases with recurrent episodes of nonaccidental trauma in children. J Trauma Acute Care Surg.

[7] Hoedeman F, Puiman PJ, Smits AW (2021). Recognition of child maltreatment in emergency departments in Europe: Should we do better?. PLoS ONE.

[8] Sittig JS, Uiterwaal CSPM, Moons KGM (2016). Value of systematic detection of physical child abuse at emergency rooms: a cross-sectional diagnostic accuracy study. BMJ Open.

[9] Louwers ECFM, Korfage IJ, Affourtit MJ (2011). Detection of child abuse in emergency departments: a multi-centre study. Arch Dis Child.

[10] Teeuw AH, Kraan RBJ, van Rijn RR (2019). Screening for child abuse using a checklist and physical examinations in the emergency department led to the detection of more cases. Acta Paediatr.

[11] Guenther E, Knight S, Olson LM (2009). Prediction of child abuse risk from emergency department use. J Pediatr.

[12] Louwers ECFM, Korfage IJ, Affourtit MJ (2012). Effects of systematic screening and detection of child abuse in emergency departments. Pediatrics.

[13] Woodman J, Lecky F, Hodes D (2010). Screening injured children for physical abuse or neglect in emergency departments: a systematic review. Child Care Health Dev.

[14] Schouten MCM, Sittig JS, Putte EM (2019). Screening op kindermishandeling: wat levert het op?. Ned Tijdschr Geneeskd.

[15] Louwers ECFM, Korfage IJ, Affourtit MJ (2012). Facilitators and barriers to screening for child abuse in the emergency department. BMC Pediatr.

[16] Tiyyagura G, Gawel M, Koziel JR (2015). Barriers and Facilitators to Detecting Child Abuse and Neglect in General Emergency Departments. Ann Emerg Med.

[17] Alink L, Prevoo M, Berkel S (2019). NPM-2017: Netherlands’ prevalence study on maltreatment of children and youth.

[18] van Berkel SR, Prevoo MJL, Linting M (2020). Prevalence of child maltreatment in the Netherlands: An update and cross-time comparison. Child Abuse Negl.

[19] van de PE, Russel IMB, Teeuw AH (2024). Medical handbook child maltreatment.

[20] KNMG (2019). Field standard on child abuse and domestic violence for hospitals.

[21] KNMG (2023). KNMG-reportingcode child abuse and domestic violence.

[22] MO Groep, Jeugdzorg (2010). Child abuse counselling and reporting centres overview 2009.

[23] CBS (2024). StatLine - open data CBS. https://opendata.cbs.nl/statline/#/CBS/nl/dataset/84847NED/table?ts=1733856454489.

[24] Health and youth I (2024). Improvement targets msz 2024 and monitoring questions.

[25] Hoedeman F, Puiman PJ, van den Heuvel EAL (2023). A validated Screening instrument for Child Abuse and Neglect (SCAN) at the emergency department. Eur J Pediatr.

[26] Fleuren MAH, Paulussen TGWM, Van Dommelen P (2014). Towards a measurement instrument for determinants of innovations. Int J Qual Health Care.

[27] Verberne LM, Kars MC, Schepers SA (2018). Barriers and facilitators to the implementation of a paediatric palliative care team. BMC Palliat Care.

[28] World Health Organization (2022). Responding to child maltreatment: a clinical handbook for health professionals.

[29] WHO guidelines for the health sector response to child maltreatment.

[30] Shahi N, Meier M, Reppucci ML (2024). Effect of Routine Child Physical Abuse Screening Tool on Emergency Department Efficiency. *Pediatr Emerg Care*.

[31] Rumball-Smith J, Fromkin J, Rosenthal B (2018). Implementation of routine electronic health record-based child abuse screening in General Emergency Departments. Child Abuse Negl.

[32] Louwers E, Korfage IJ, Affourtit MJ (2014). Accuracy of a screening instrument to identify potential child abuse in emergency departments. Child Abuse Negl.

[33] Suresh S, Heineman E, Meyer L (2022). Improved Detection of Child Maltreatment with Routine Screening in a Tertiary Care Pediatric Hospital. J Pediatr.

[34] Lindberg DM, Peterson RA, Orsi-Hunt R (2024). Routine Emergency Department Screening to Decrease Subsequent Physical Abuse. Ann Emerg Med.

[35] Berger RP, Pierce MC (2024). Does Routine Screening for Child Abuse Decrease Subsequent Abuse? One Big Step Forward, Two Small Steps Back. Ann Emerg Med.

