# Supplementary material for: Enhancing implementation and compliance of the Screening Instrument for Child Abuse and Neglect (SCAN) in emergency departments in the Netherlands
Source: BMJ Paediatr Open. 2026 Feb 24;10(1):e003362. doi: 10.1136/bmjpo-2025-003362 (PMC12933765; doi:10.1136/bmjpo-2025-003362)
Supplement: online supplemental file 2 [file bmjpo-10-1-s002.docx]

**Guideline for Semi-Structured Interviews Following the MIDI Analysis**

V1.0

September 2021

**Background information:**

*The interview will be conducted by a member of the research team. Participants will be selected by the clinical ambassador through purposive sampling. At a minimum, the group should include a paediatrician, the designated child abuse officer, an emergency physician, and an emergency department nurse. Preferably, physicians from other specialties (e.g. surgery, neurology) will also take part. The date of the interview will be determined by the clinical ambassador in consultation with the research team, and scheduled no earlier than two months after distribution of the MIDI.*

- **Welcome and introduction**
  - Briefly explain the purpose of the interview and how the results will be used.
  - Emphasise confidentiality and anonymity of responses.
- **Presentation of general MIDI results**
  - Number of respondents.
  - Key respondent characteristics.
- **Feedback on identified facilitators**
  - Why do you think these aspects worked well in your centre?
  - What can other centres learn from your experiences?
- **Feedback on identified barriers**
  - Why do you think these aspects were barriers in your centre?
  - What can other centres learn from your experiences?
- **Centre-Specific facilitators and barriers compared to other centres (anonymous comparison)**
- **Centre-Specific facilitators and barriers per subgroup compared to other centres**
- **Open feedback**
  - Space for any additional comments or reflections from the centre regarding SCAN&STEPS.
