# Supplementary material for: Enhancing implementation and compliance of the Screening Instrument for Child Abuse and Neglect (SCAN) in emergency departments in the Netherlands
Source: BMJ Paediatr Open. 2026 Feb 24;10(1):e003362. doi: 10.1136/bmjpo-2025-003362 (PMC12933765; doi:10.1136/bmjpo-2025-003362)
Supplement: online supplemental file 3 [file bmjpo-10-1-s003.docx]

**Supplement table 1: Subgroup analysis hospital type (academic vs. non-academic)**

|  | Academic | | | | | | Non-academic | | | | |  |
| --- | --- | --- | --- | --- | --- | --- | --- | --- | --- | --- | --- | --- |
| MIDI item | **N** | **Mean (SD)** | **Disagree / totally disagree (%)** | **Neutral (%)** | | **Agree / totally agree (%)** | **N** | **Mean (SD)** | **Disagree / totally disagree (%)** | **Neutral (%)** | **Agree / totally agree (%)** | **Chi2** |
| 1.Procedural clarity | 60 | 3.63 (0.66) | 6.7 | | 26.7 | 66.6 | 132 | 3.78 (0.71) | 6.1 | 13.6 | 80.3 | 0.12 |
| 2.Completeness | 58 | 3.53 (0.71) | 8.6 | | 32.8 | 58.6 | 127 | 3.70 (0.62) | 4.7 | 24.4 | 70.8 | 0.40 |
| 3.Complexity* | 58 | 3.52 (0.92) | 55.2 | | 31 | 13.8 | 126 | 3.82  (0.92) | 67.5 | 23.8 | 8.7 | 0.33 |
| 4.Compatibility | 58 | 3.43 (0.82) | 10.3 | | 36.2 | 53.4 | 125 | 3.62  (0.72) | 8.8 | 22.4 | 68.8 | 0.19 |
| 5.Relevance to patient | 57 | 3.65 (0.61) | 3.5 | | 31.6 | 64.9 | 123 | 3.81  (0.66) | 5.7 | 13 | 81.3 | 0.05 |
| 6a.Personal advantages/disadvantages - actions | 55 | 3.27 (0.89) | 20 | | 36.4 | 43.7 | 113 | 3.43  (0.81) | 11.5 | 37.2 | 51.3 | 0.62 |
| 6b. Personal advantages/disadvantages - documentation | 55 | 3.47 (0.63) | 7.3 | | 38.2 | 54.5 | 112 | 3.34  (0.73) | 10.7 | 46.4 | 42.9 | 0.34 |
| 7a.Outcome expectations - recognition | 55 | 3.85 (0.71) | 3.6 | | 21.8 | 74.5 | 110 | 3.75  (0.73) | 5.5 | 22.7 | 71.8 | 0.87 |
| 7b.Outcome expectations – expectance | 54 | 3.22 (0.66) | 9.3 | | 63 | 27.8 | 109 | 3.31  (0.70) | 10.1 | 52.3 | 37.6 | 0.59 |
| 8.Perception of responsibility | 54 | 4.20 (0.71) | 1.9 | | 11.1 | 87.1 | 109 | 4.26  (0.75) | 2.7 | 7.3 | 89.9 | 0.87 |
| 9.Social support | 54 | 4.19 (0.65) | 13 | | 55.6 | 31.5 | 109 | 3.98  (0.68) | 2.8 | 15.6 | 81.6 | 0.24 |
| 10a.Self-efficacy – SCAN | 53 | 4.02 (0.60) | 0 | | 17 | 83.1 | 109 | 4.16  (0.61) | 0.9 | 6.4 | 92.7 | 0.16 |
| 10b.Self-efficacy – STEPS | 53 | 3.79 (0.72) | 3.8 | | 26.4 | 69.8 | 109 | 3.62  (0.73 | 5.5 | 30.3 | 64.2 | 0.43 |
| 11.Knowledge | 53 | 3.87 (0.65) | 1.9 | | 22.6 | 75.5 | 108 | 3.86  (0.68) | 4.6 | 13.9 | 81.5 | 0.52 |
| 12.Formal ratification by management^#^ | 52 | 1.85 (0.36) | 15.4 | |  | 84.6 | 107 | 1.87  (0.34) | 13.1 |  | 86.9 | 0.69 |
| 13.Staff capacity | 52 | 3.52 (0.78) | 9.6 | | 36.5 | 53.9 | 105 | 3.67  (0.63) | 4.8 | 24.8 | 70.5 | 0.04 |
| 14.Availablility of materials and resources | 52 | 3.60 (0.57) | 1.9 | | 38.5 | 59.6 | 105 | 3.76  (0.63) | 3.8 | 20 | 76.2 | 0.14 |
| 15.Unsettled organisation^#^* | 53 | 1.43 (0.50) | 56.6 | |  | 43.3 | 104 | 1.61  (0.49) | 43.4 |  | 60.6 | 0.04 |
| 16.Acces to information on SCAN&STEPS tools use | 53 | 3.85 (0.66) | 1.9 | | 24.5 | 73.6 | 104 | 3.78  (0.61) | 2.9 | 20.2 | 76.9 | 0.31 |
| 17.Legislations and regulations | 51 | 3.76 (0.71) | 3.9 | | 27.5 | 68.7 | 104 | 3.93 (0.61) | 1.9 | 16.3 | 81.8 | 0.33 |

**Supplement table 2: Subgroup analysis work experience**

|  | 0-5 years | | | | | 6-15 years | | | | | >15 years | | | | |  |
| --- | --- | --- | --- | --- | --- | --- | --- | --- | --- | --- | --- | --- | --- | --- | --- | --- |
| MIDI item | **N** | **Mean (SD)** | **disagree / totally disagree** | **neutral** | **agree / totally agree** | **N** | **Mean (SD)** | **disagree / totally disagree** | **neutral** | **agree / totally agree** | **N** | **Mean (SD)** | **disagree / totally disagree** | **neutral** | **agree / totally agree** | **Chi2** |
| 1.Procedural clarity | 59 | 3.76 (0.77) | 6.8 | 9.1 | 84.1 | 87 | 3.82 (0.60) | 4.1 | 13.7 | 82.2 | 67 | 3.70 (0.71) | 5.4 | 17.9 | 76.8 | 0.34 |
| 2.Completeness | 53 | 3.66 (0.78) | 11.4 | 15.9 | 72.7 | 87 | 3.69 (0.60) | 4.1 | 23.3 | 72.6 | 65 | 3.60 (0.66) | 7.1 | 25 | 67.9 | 0.21 |
| 3.Complexity* | 52 | 3.85 (0.85) | 68.2 | 25 | 6.8 | 87 | 3.71 (0.94) | 67.1 | 19.2 | 13.7 | 65 | 3.74 (0.92) | 67.8 | 21.4 | 10.7 | 0.70 |
| 4.Compatibility | 52 | 3.62 (0.72) | 6.8 | 20.5 | 72.8 | 86 | 3.57 (0.73) | 9.3 | 24.7 | 65.9 | 65 | 3.54 (0.79) | 10.7 | 28.6 | 60.7 | 0.54 |
| 5.Relevance to patient | 50 | 3.76 (0.74) | 6.8 | 13.6 | 79.5 | 86 | 3.81 (0.58) | 2.7 | 21.9 | 75.3 | 64 | 3.75 (0.64) | 5.4 | 16.1 | 78.6 | 0.32 |
| 6a.Personal advantages/disadvantages - actions | 45 | 3.58 (0.89) | 13.6 | 22.7 | 63.6 | 82 | 3.35 (0.81) | 16.4 | 34.2 | 49.3 | 61 | 3.33 (0.81) | 10.7 | 46.4 | 42.9 | 0.05 |
| 6b. Personal advantages/disadvantages - documentation | 45 | 3.33 (0.83) | 15.9 | 36.4 | 47.8 | 81 | 3.43 (0.71) | 9.6 | 41.1 | 49.3 | 60 | 3.42 (0.70) | 7.1 | 42.9 | 50 | 0.69 |
| 7a.Outcome expectations - recognition | 45 | 4.00 (0.67) | 2.3 | 15.9 | 81.9 | 80 | 3.74 (0.65) | 5.5 | 21.9 | 72.6 | 59 | 3.76 (0.80) | 5.4 | 19.6 | 75 | 0.72 |
| 7b.Outcome expectations – expectance | 45 | 3.29 (0.70) | 9.1 | 59.1 | 31.8 | 78 | 3.28 (0.66) | 9.6 | 56.2 | 34.3 | 58 | 3.33 (0.74) | 10.7 | 51.8 | 37.5 | 0.99 |
| 8.Perception of responsibility | 45 | 4.22 (0.70) | 2.3 | 9.1 | 88.7 | 78 | 4.32 (0.67) | 1.4 | 6.8 | 91.7 | 58 | 4.21 (0.79) | 3.6 | 5.4 | 91.1 | 0.92 |
| 9.Social support | 45 | 4.27 (0.75) | 2.3 | 11.4 | 86.4 | 78 | 3.92 (0.68) | 4.1 | 15.1 | 80.9 | 58 | 4.00 (0.56) | 0 | 14.3 | 85.7 | 0.68 |
| 10a.Self-efficacy – SCAN | 44 | 4.16 (0.65) | 0 | 13.6 | 86.3 | 78 | 4.12 (0.62) | 1.4 | 5.5 | 93.1 | 58 | 4.09 (0.51) | 0 | 7.1 | 92.9 | 0.50 |
| 10b.Self-efficacy – STEPS | 44 | 3.77 (0.68) | 4.5 | 22.7 | 72.7 | 78 | 3.73 (0.72) | 4.1 | 24.7 | 71.2 | 58 | 3.57 (0.75) | 5.4 | 37.5 | 57.1 | 0.24 |
| 11.Knowledge | 44 | 3.86 (0.59) | 4.5 | 11.4 | 84.1 | 78 | 3.85 (0.72) | 5.5 | 13.7 | 80.9 | 57 | 3.91 (0.58) | 0 | 19.6 | 80.4 | 0.27 |
| 12.Formal ratification by management^#^ | 44 | 1.89 (0.32) | 11.4 |  | 88.6 | 76 | 1.87 (0.34) | 12.3 |  | 87.7 | 57 | 1.86 (0.35) | 14.3 |  | 85.7 | 0.79 |
| 13.Staff capacity | 44 | 3.66 (0.65) | 4.5 | 29.5 | 65.9 | 75 | 3.55 (0.72) | 8.2 | 28.8 | 63 | 56 | 3.70 (0.66) | 5.4 | 25 | 69.7 | 0.96 |
| 14.Availablility of materials and resources | 44 | 3.73 (0.59) | 2.3 | 27.3 | 70.4 | 75 | 3.60 (0.68) | 5.5 | 30.1 | 64.3 | 56 | 3.84 (0.53) | 1.8 | 17.9 | 80.4 | 0.47 |
| 15.Unsettled organisation^#^* | 44 | 1.66 (0.48) | 65.9 |  | 34.1 | 75 | 1.49 (0.50) | 50.7 |  | 49.3 | 56 | 1.55 (0.50) | 55.4 |  | 44.6 | 0.49 |
| 16.Acces to information on SCAN&STEPS tools use | 44 | 3.98 (0.51) | 0 | 13.6 | 86.4 | 75 | 3.76 (0.59) | 1.4 | 24.7 | 73.9 | 56 | 3.77 (0.69) | 5.4 | 21.4 | 73.2 | 0.10 |
| 17.Legislations and regulations | 44 | 3.84 (0.57) | 2.3 | 18.2 | 79.5 | 73 | 4.01 (0.61) | 0 | 17.8 | 82.2 | 56 | 3.79 (0.71) | 5.4 | 21.4 | 73.2 | 0.38 |
