# Supplementary material for: Enhancing implementation and compliance of the Screening Instrument for Child Abuse and Neglect (SCAN) in emergency departments in the Netherlands
Source: BMJ Paediatr Open. 2026 Feb 24;10(1):e003362. doi: 10.1136/bmjpo-2025-003362 (PMC12933765; doi:10.1136/bmjpo-2025-003362)
Supplement: Supplementary file 4 [file bmjpo-2025-003362.reviewer_comments.pdf]

## PEER REVIEW HISTORY

BMJ Paediatrics Open publishes all reviews undertaken for accepted manuscripts. Reviewers are asked to complete a checklist review form and are provided with free text boxes to elaborate on their assessment. These free text comments are reproduced below.

## ARTICLE DETAILS

| | |
|---|---|
| **TITLE (PROVISIONAL)** | Enhancing implementation and compliance of the Screening instrument for Child Abuse and Neglect (SCAN) in Emergency Departments in the Netherlands |
| **AUTHORS** | Heuvel, Eline A.L. van den<br>Vries, D.S de<br>Jong-van Kempen, B.M. de<br>Bakx, R.<br>Bos, R.F.<br>van Empelen, P.<br>Hoedeman, F.<br>Kappen, T.H.<br>Russel-Kampschoer, I.M.B.<br>Puiman, P.<br>Schouten, M.C.M.<br>Teeuw, Rian<br>Zwaans, C.J.<br>Nijhof, Sanne L.<br>van de Putte, Elise M. |

## VERSION 1 – REVIEW

| | |
|---|---|
| **REVIEWER NAME** | Otterman, Gabriel |
| **REVIEWER AFFILIATION** | Uppsala University Department of Womens and Childrens Health, Uppsala University Children's Hospital |
| **REVIEWER CONFLICT OF INTEREST** | No Conflict of Interest |
| **AI DISCLOSURE** | I have used Microsoft Co-pilot in text editing in accordance with my institutional license and guidance. The conceptual and scientific content is entirely my own. Searches in the grey literature were conducted on Perplexity. |
| **DATE REVIEW RETURNED** | 07-Mar-2025 |

| | |
|---|---|
| **GENERAL COMMENTS** | Many thanks for the invitation to review the manuscript "Towards Uniform Recognition of Child Abuse in the Netherlands: Implementing the Screening Instrument for Child Abuse and Neglect (SCAN)" submitted for publication in BMJ Paediatrics Open. The authors address a critical issue in child maltreatment recognition within EDs in the Netherlands, a topic of significant relevance to healthcare professionals, child welfare agencies and policy makers. Given the well-documented challenges in the early detection of and responses to child maltreatment, particularly in healthcare sector settings, the study’s focus on the implementation and evaluation of SCAN is timely and important with valuable learning for other countries.<br><br>It was a pleasure to read this manuscript; it is well-crafted and written in clear, accessible language. The tables and figures are well-constructed for readability, effectively presenting the findings. |

| | |
|---|---|
| | As the analysis relies on descriptive statistics, the methods are straightforward and generally well-developed. These strengths allow me to focus this review on broader conceptual and methodological considerations. I offer several suggestions—listed without a particular order of priority—that I hope will help the authors further strengthen the manuscript. I recognise that addressing these comments within the constraints of the word limit may be challenging, and I hope the editorial team will allow some flexibility given the pivotal nature of this research.<br><br>The title of the paper and stated aims of the study could be more precisely aligned with the actual focus of the research. The authors describe their aim as evaluating compliance with screening in the ED. However, the study does more than assess compliance rates—it examines whether compliance (the proportion of eligible children in the ED who are screened) can be improved through the implementation of a range of support tools. These include integration into the EHR, training, clinical ambassadors, pocket cards, dissemination efforts, and other strategies. The aims should be reformulated to reflect this broader implementation-focused evaluation.<br><br>Consider, for example, the title "Enhancing the Implementation and Compliance of the SCAN Screening Instrument for Child Maltreatment in Emergency Departments in the Netherlands" if this better reflects the focus of the study.<br><br>The terminology used to describe SCAN, and its components could be clarified. The distinction between SCAN screening and SCAN (SCAN screening + SCAN-FU) is difficult to follow throughout the manuscript.<br><br>The rationale for the study is well established, highlighting the apparent decline in the recognition of child maltreatment in Dutch EDs. However, the introduction could better clarify why SCAN was chosen as the intervention. While the authors note that existing screening instruments such as ESCAPE and SPUTOVAMO have been shown to improve detection rates, the manuscript does not fully explain why SCAN was developed instead of adapting these existing tools. Additionally, the nine hospital EDs studied had some form of screening instrument in place at baseline before the implementation of SCAN screening. The specific instruments used at baseline should be indicated, (Table 2) to provide a clearer picture of the starting point for each ED, which will allow better interpretation of the compliance rates for SCAN.<br><br>The chain of care for child protection in the Netherlands is complex may be difficult to follow for international readers, particularly those practicing in systems with statutory child protection agencies that have enforcement roles. It is essential to clarify that the Netherlands does not operate under a mandatory reporting system in which healthcare professionals directly report to statutory agencies. The authors should provide more context—perhaps in an insert—on the KNMG medical reporting law, outlining its key provisions and how it shapes the SCAN-FU component. A crucial point requiring further elaboration is the discretion physicians appear to have in deciding whether to refer cases to Safe at Home or to manage concerns within the healthcare system. This discretionary process is unique compared to many other jurisdictions with mandatory reporting for healthcare professionals and warrants further description. |

| | |
|---|---|
| | Additionally, the manuscript should provide a clearer overview of the full chain of care, including the role of Safe at Home, which apparently does not have compulsory intervention levers at its disposal. It is unclear for the reader how Safe at Home substantiates referrals received from healthcare professionals, and this should be further explained. It would be valuable to explicitly describe how referrals from healthcare professionals are processed, the threshold for escalation to the Child Care and Protection Board or law enforcement, and the circumstances under which a child may be placed in care. This structure differs significantly from systems and countries in which a statutory CPS agencies possess a range of voluntary and compulsory supportive interventions.<br><br>The classification of hospitals as academic, teaching, or general should be further explained to help international readers contextualise the study setting. Healthcare systems vary widely between countries, and these distinctions may influence screening practices, resource availability, and institutional policies. Providing a brief description of how these hospital types are distinct within the Dutch healthcare system would be helpful.<br>Additionally, in the analysis, hospital subgroups were defined, but the observed differences between hospital types and their approach to medical reporting were not explained or discussed. Related to this, was any data collected on the subspecialties of physicians using SCAN-FU? Understanding whether screening and follow-up behaviours differed among clinicians in paediatric surgery, emergency medicine, paediatrics, or psychiatry could provide valuable insights into implementation and potential barriers to compliance.<br><br>The authors appear to have used a sequential explanatory mixed methods approach to examine the usability of the SCAN instrument, incorporating qualitative data to complement and following the MIDI appraisal. However, the manuscript does not specify a methodological framework for the qualitative component of the study. Clarifying whether a specific qualitative method—e.g., thematic analysis or content analysis—was used would strengthen the rigour of the study and bring the respondent quotes into context. The authors should explicitly state the mixed methods design in the methods section and provide the interview guide, the selection or sampling of the interviewees and relevant coding framework in the supplemental material to allow for reproducibility and improve transparency.<br><br>The term "suspected child maltreatment" should be replaced with "concerns for child maltreatment", e.g., Figure 1 and elsewhere the manuscript where applicable. The word "suspicion" can imply intentional harm or concealment by caregivers, whereas "concerns for child maltreatment" is a more neutral and appropriate term within a safeguarding framework. This phrasing better reflects the early identification and screening process, ensuring that concerns are acknowledged without implying intent or any level of certainty.<br><br>Finally, and perhaps most importantly, the authors should be more explicit in the discussion about the lack of evidence supporting universal screening for child maltreatment. The WHO does not support universal screening for child maltreatment in the healthcare sector. Instead, WHO recommends a case-finding approach for identifying children exposed to maltreatment (e.g., Responding to |

| | |
|---|---|
| | Child Maltreatment: A Clinical Handbook for Health Professionals, 2022). This is as there is a lack of substantial evidence that early detection through universal screening improves outcomes for children and their families.<br><br>The Dutch LECK network is known for applying a Bayesian approach to child maltreatment evaluations (van Rijn et al., BMJ Open, 2019). However, there is insufficient understanding of the positive and negative predictive values of the SCAN instrument, which is critical for assessing its diagnostic utility. Without data on key characteristics and baseline prevalence in the studied population, it is not possible to calculate likelihood ratios, making it difficult to determine how well SCAN differentiates between true and false cases of concern. Given that screening in a low-prevalence population by definition carries a significant risk of false positives, further analysis may be necessary to evaluate the instrument's performance and its implications for clinical decision-making.<br><br>Case-finding approaches is exemplified in the method used by Lindberg et al. (ref 30, 31 in the manuscript), which selectively identifies a population with a higher baseline prevalence of maltreatment building on evidence-based clinical decision rules and specific presenting conditions (e.g., manifest injuries in children under six years). These distinctions are important in evaluating the feasibility, sensitivity, and real-world applicability of different screening strategies in emergency care.<br><br>It is crucial to be explicit about the available interventions for children and families identified through screening. When screening for any disease or condition, it is important to ensure that a clear intervention treatment or management pathway is defined for those who screen positive in order to begin to understand whether an intervention ultimately leads to improved outcomes for the child. As understood, Dutch healthcare professionals have an obligation to arrange help for families when there are concerns for child maltreatment.<br><br>This concern is further compounded by the lack of clarity regarding what happens after a child screens positive—whether the intervention involves referral to Safe at Home, follow up healthcare services, or further interventions in the child protection chain of care. Without clear evidence of fidelity in the decision point physician referral to Safe at Home / retention in healthcare decision tree, or data to otherwise demonstrate improved outcomes, the discussion should acknowledge these critical gaps and frame SCAN's implementation within the broader debate on the effectiveness and risks of universal child maltreatment screening.<br><br>These issues are especially important when clinicians—encouraged by the published validation of a tool like SCAN—adapt it to different legislative and policy contexts. For example, SCAN is currently being piloted in Sweden, where robust mandatory reporting laws – to my understanding -- mean that a child who checked positive on CAST screening at an ED triage would automatically trigger a CPS report. Such differences in child protection systems can significantly alter the consequences of screening, reinforcing the need to carefully evaluate predictive values, unintended consequences, and system capacity before broader implementation. The discussion section should include a stronger statement of caution and limitations, acknowledging the lack of evidence linking universal |

| | |
|---|---|
| | screening to improved outcomes, the absence of predictive validity data, and the risk of false positives in a low-prevalence population. The discussion does not currently consider how SCAN functions in diverse policy environments and systems, which is critical for its adaptation elsewhere. A more explicit discussion of these issues would ensure that policymakers and clinicians interpret the findings with appropriate caution and recognise the need for further validation before wider adoption. |

**VERSION 1 – AUTHOR RESPONSE**

Comment 1:

> *Many thanks for the invitation to review the manuscript "Towards Uniform Recognition of Child Abuse in the Netherlands: Implementing the Screening Instrument for Child Abuse and Neglect (SCAN)" submitted for publication in BMJ Paediatrics Open. The authors address a critical issue in child maltreatment recognition within EDs in the Netherlands, a topic of significant relevance to healthcare professionals, child welfare agencies and policy makers. Given the well-documented challenges in the early detection of and responses to child maltreatment, particularly in healthcare sector settings, the study's focus on the implementation and evaluation of SCAN is timely and important with valuable learning for other countries. It was a pleasure to read this manuscript; it is well-crafted and written in clear, accessible language. The tables and figures are well-constructed for readability, effectively presenting the findings. As the analysis relies on descriptive statistics, the methods are straightforward and generally well-developed. These strengths allow me to focus this review on broader conceptual and methodological considerations. I offer several suggestions—listed without a particular order of priority—that I hope will help the authors further strengthen the manuscript. I recognise that addressing these comments within the constraints of the word limit may be challenging, and I hope the editorial team will allow some flexibility given the pivotal nature of this research.*

Response to comment 1:

We would like to sincerely thank dr. Otterman for his thoughtful and encouraging evaluation of our manuscript. We are pleased to hear that the topic, writing style, and presentation of our findings were well received. We also appreciate the recognition of the broader relevance of our work.

We are grateful for the constructive suggestions and have carefully considered each of them in revising our manuscript. Where possible, we have incorporated the recommendations in a manner consistent with the scope of the paper, which has unavoidably resulted in a modest word limit exceedance of 242 words.

Revision in text: NA

Comment 2:

> *The title of the paper and stated aims of the study could be more precisely aligned with the actual focus of the research. The authors describe their aim as evaluating compliance with screening in the ED. However, the study does more than assess compliance rates—it examines whether compliance (the proportion of eligible children in the ED who are screened) can be improved through the implementation of a range of support tools. These include integration into the EHR, training, clinical ambassadors, pocket cards, dissemination efforts, and other strategies. The aims should be reformulated to reflect this broader implementation-focused evaluation.*

*Consider, for example, the title “Enhancing the Implementation and Compliance of the SCAN Screening Instrument for Child Maltreatment in Emergency Departments in the Netherlands” if this better reflects the focus of the study.*

Response to comment 2:

We gratefully accept the reviewer's suggestion for the adjustment of the title to ensure it more accurately reflects the present focus and stage of the study. We have reformulated the aims to better reflect the implementation-focused evaluation.

*Revision in title*: Enhancing implementation and compliance of the Screening instrument for Child Abuse and Neglect (SCAN) in Emergency Departments in the Netherlands.

*Revision in abstract*: This study examines the implementation of the Screening Instrument for Child Abuse and Neglect (SCAN) and the Structured Tool for Evaluating Positive Screened cases (STEPS) in nine Emergency Departments (EDs). The study aimed to assess whether implementation could enhance compliance with SCAN and to evaluate the usability of SCAN&STEPS within an implementation-focused design, in response to the decline in child maltreatment recognition observed in Dutch EDs over the past decade.

**METHODS**

SCAN&STEPS was embedded in ED-workflow and electronic health records (EHRs), supported by a selection of implementation strategies, including e-learning, policy manuals, and an awareness campaign. Effectiveness was assessed through compliance with SCAN, comparing pre- and post-implementation screening rates, with a ≥10% increase defined as clinically relevant. Usability of SCAN&STEPS was examined using a mixed-method design combining the Measurement Instrument for Determinants of Innovations and semi-structured interviews. Subgroup analyses were conducted by hospital type, EHR, profession, and years of working experience.

*Revision in text*: This study aims to evaluate whether the implementation of SCAN in the emergency department of 9 Dutch hospitals can improve compliance and thereby strengthen the recognition of child maltreatment concerns, within an implementation-focused evaluation. To support subsequent medical diagnostic follow-up and guidance in any safety concerns, the Structured Tool for Evaluating Positive Screened cases (STEPS) provides physicians with standardised guidance for navigating the medical reporting code. The second aim for this study is to evaluate the usability of the SCAN&STEPS in daily practice. The results are intended to support the nationwide implementation of the SCAN&STEPS, with the overall goal of improving the early identification of child maltreatment.

Comment 3:

*The terminology used to describe SCAN, and its components could be clarified. The distinction between SCAN screening and SCAN (SCAN screening + SCAN-FU) is difficult to follow throughout the manuscript.*

Response to comment 3:

Thank you for this valuable comment. To improve clarity, we have revised the terminology throughout the manuscript. What was previously referred to as “SCAN” and “SCAN-FU” is now consistently described as:

1. SCAN: the universal screening in the nursing record that appears for all patients (former SCAN screening)
2. STEPS (Structured Tool for Evaluating Positive Screened cases): the decision support module in the physician's record, activated for SCAN-positive cases (former SCAN-FU).
3. The full protocol is named SCAN&STEPS

We believe this clearer terminology improves readability and reduces potential confusion.

*Revision in text*: throughout the manuscript we have implemented this revised terminology.

Comment 4:

> *The rationale for the study is well established, highlighting the apparent decline in the recognition of child maltreatment in Dutch EDs. However, the introduction could better clarify why SCAN was chosen as the intervention. While the authors note that existing screening instruments such as ESCAPE and SPUTOVAMO have been shown to improve detection rates, the manuscript does not fully explain why SCAN was developed instead of adapting these existing tools. Additionally, the nine hospital EDs studied had some form of screening instrument in place at baseline before the implementation of SCAN screening. The specific instruments used at baseline should be indicated, (Table 2) to provide a clearer picture of the starting point for each ED, which will allow better interpretation of the compliance rates for SCAN.*

Response to comment 4:

Thank you for this important observation. We have clarified in the introduction that SCAN was developed to address the lack of a uniform, feasible, and validated instrument, as existing tools (ESCAPE, SPUTOVAMO, SPUTOVAMO-R) showed variability in validity across prior studies. In addition, Table 2 has been updated to specify the screening instruments used at baseline in each of the nine emergency departments.

*Revision in text*: Although the prevalence of child abuse remained stable, reports to Safe at Home by hospital-based healthcare professionals have declined [22,23]. This suggests reduced recognition of possible maltreatment, despite mandatory staff training on child abuse recognition mandated by the Inspectorate of Health [24]. A possible contributing factor may be the lack of a uniform, feasible and validated screening instrument, as existing tools (e.g. ESCAPE, SPUTOVAMO) showed variability in usability, content and validity. This resulted in variable maltreatment recognition protocols and inconsistent hospital policies and performance. These inconsistencies hinder inter-hospital comparability, limiting opportunities for shared learning and system-wide healthcare improvement. To address this hiatus, the Screening instrument for Child Abuse and Neglect (Figure 2B) was developed as a brief, uniform and validated screening instrument. SCAN was derived from the data from three large validation studies of SPUTOVAMO, SPUTOVAMO-R and ESCAPE (after harmonization of questions and outcome) and uses the most discriminative questions to improve validity and to ensure maximal feasibility and adherence in daily ED practice [25].

*Revision in tables:* Table 2 has been updated to include the screening instruments that were in place at baseline in each of the nine emergency departments, providing a clearer overview of the starting point for SCAN implementation.

Comment 5:

*The chain of care for child protection in the Netherlands is complex may be difficult to follow for international readers, particularly those practicing in systems with statutory child protection agencies that have enforcement roles. It is essential to clarify that the Netherlands does not operate under a mandatory reporting system in which healthcare professionals directly report to statutory agencies. The authors should provide more context—perhaps in an insert—on the KNMG medical reporting law, outlining its key provisions and how it shapes the SCAN-FU component. A crucial point requiring further elaboration is the discretion physicians appear to have in deciding whether to refer cases to Safe at Home or to manage concerns within the healthcare system. This discretionary process is unique compared to many other jurisdictions with mandatory reporting for healthcare professionals and warrants further description. Additionally, the manuscript should provide a clearer overview of the full chain of care, including the role of Safe at Home, which apparently does not have compulsory intervention levers at its disposal. It is unclear for the reader how Safe at Home substantiates referrals received from healthcare professionals, and this should be further explained. It would be valuable to explicitly describe how referrals from healthcare professionals are processed, the threshold for escalation to the Child Care and Protection Board or law enforcement, and the circumstances under which a child may be placed in care. This structure differs significantly from systems and countries in which a statutory CPS agencies possess a range of voluntary and compulsory supportive interventions.*

Response to comment 5:

We thank the reviewer for highlighting this important point. In response, we have included a description of the Dutch child protection system, with specific reference to the Domestic Violence and Child Abuse Reporting Code Act and the considerations that physicians may take into account within this framework (illustrated in Figure 1). Furthermore, we have clarified the absence of statutory mandatory reporting and outlined the full chain of care in the Netherlands in the text. We believe that these additions enhance the clarity of the manuscript for an international readership and place the use of a screening tool for child abuse in the context of the Dutch situation.

*Revision in text*: Over the past decade, the estimated prevalence of child maltreatment in the Netherlands remained unchanged [17,18]. In cases with concerns for child maltreatment, Dutch physicians do not have statutory duty to report, but they are required to follow the mandatory reporting code. This code provides a structured framework for deciding whether concerns should be referred to '*Safe at Home',* the national centre for advice and reporting on concerns for child abuse and interpersonal violence (Figure 1: Dutch child protection system) [19–21].

Comment 6:

*The classification of hospitals as academic, teaching, or general should be further explained to help international readers contextualise the study setting. Healthcare systems vary widely between countries, and these distinctions may influence screening practices, resource availability, and institutional policies. Providing a brief description of how these hospital types are distinct within the Dutch healthcare system would be helpful.*
*Additionally, in the analysis, hospital subgroups were defined, but the observed differences between hospital types and their approach to medical reporting were not explained or discussed. Related to this, was any data collected on the subspecialties of physicians using SCAN-FU? Understanding whether screening and follow-up behaviours differed among clinicians in paediatric surgery, emergency medicine, paediatrics, or psychiatry could provide valuable insights into implementation and potential barriers to compliance.*

Response to comment 6:

We thank the reviewer for this thoughtful and constructive comment. In the Netherlands, all hospitals are bound to uniform national guidelines for child abuse screening and reporting. For this reason, screening and reporting procedures are comparable across institutions, irrespective of hospital type. But we agree that international readers may benefit from clarification regarding hospital types. To enhance clarity and international recognisability, we therefore chose to distinguish only between *university* and *non-university* hospitals, which is also in line with our subgroup analyses. This clarification has now been added to the methods section.

*Revision in text*: To ensure broad applicability of the SCAN&STEPS, a purposeful sample of hospitals was selected, including 3 university hospitals, and 6 non-university hospitals. These 9 hospitals, collectively operating 12 ED locations are all hospital-based and provided care for patients under 18 years (among other age groups).

*Hospital type*:

Subgroup analysis across hospital types identified two significant differences (see Supplement C). First, professionals in non-university hospitals more often considered their department as sufficiently staffed to work with the SCAN&STEPS, although this was not deemed a facilitator, as agreement did not reach the predefined threshold (>80%). Second, professionals in university hospitals more frequently reported organizational changes affecting implementation. While the in-depth interviews did not provide concrete explanations, these findings may reflect that academic hospitals are typically more dynamic environments due to training responsibilities and research activities, which can lead to greater organizational changes and higher staff turnover. Combined with reported perceptions of fewer staff resources, this may ultimately pose challenges to consistent compliance.

Comment 7:

> *The authors appear to have used a sequential explanatory mixed methods approach to examine the usability of the SCAN instrument, incorporating qualitative data to complement and following the MIDI appraisal. However, the manuscript does not specify a methodological framework for the qualitative component of the study. Clarifying whether a specific qualitative method—e.g., thematic analysis or content analysis—was used would strengthen the rigour of the study and bring the respondent quotes into context. The authors should explicitly state the mixed methods design in the methods section and provide the interview guide, the selection or sampling of the interviewees and relevant coding framework in the supplemental material to allow for reproducibility and improve transparency.*

Response to comment 7:

We thank the reviewer for this constructive comment. In the revised manuscript, we have clarified the mixed methods design, in which the interviews were conducted to complement and further interpret the findings of the MIDI appraisal. To enhance transparency and reproducibility, we have added the semi-structured interview guide (Supplement B), detailed the purposive sampling strategy, and described the **descriptive, deductive content summarisation procedure** that was applied to the interview material. In this approach, **centre-level summaries of facilitators and barriers were generated by the research team, drawing on the hospital-specific MIDI findings as a deductive framework. Draft summaries were reviewed and refined in team discussions to ensure consistency and minimise bias.** We believe these clarifications **improve transparency and strengthen the methodological rigour of the study, while remaining aligned with the pragmatic, comparative aim of this qualitative strand.**

*Revision in text:* Subsequently, the clinical ambassador at each hospital purposively selected a smaller, representative sample of SCAN&STEPS users, including at least a paediatrician, an emergency department physician and an emergency department nurse with whom semi-structured in-depth interviews were conducted. An interview guide was developed by the research team to ensure consistency (see Supplement B). These interviews, conducted in hybrid format via Microsoft Teams and at least two months after distribution of the MIDI-questionnaire, followed guided questions informed by the hospital-specific MIDI results and explored facilitators and barriers in greater detail. Findings were analysed using **a descriptive, deductive content summarisation approach, in which responses were categorised as facilitators or barriers and summarised at the centre level. Draft summaries were reviewed within the research team to ensure consistency.** A formal thematic analysis was not conducted, as the study aimed to capture centre-specific facilitators and barriers for structured comparison across sites rather than to develop new themes or theoretical constructs.

Comment 8:

> *The term "suspected child maltreatment" should be replaced with "concerns for child maltreatment", e.g., Figure 1 and elsewhere the manuscript where applicable. The word "suspicion" can imply intentional harm or concealment by caregivers, whereas "concerns for child maltreatment" is a more neutral and appropriate term within a safeguarding framework. This phrasing better reflects the early identification and screening process, ensuring that concerns are acknowledged without implying intent or any level of certainty.*

Response to comment 8:

We thank the reviewer for this valuable suggestion. In the revised manuscript, we have replaced the term *'suspected child maltreatment'* with *'concerns for child maltreatment'* in Figure 1 and throughout all relevant sections. This revised terminology better conveys the status of the screening process and aligns with safeguarding principles, thereby avoiding unintended implications regarding intent or certainty

Comment 9

> *Finally, and perhaps most importantly, the authors should be more explicit in the discussion about the lack of evidence supporting universal screening for child maltreatment. The WHO does not support universal screening for child maltreatment in the healthcare sector. Instead, WHO recommends a case-finding approach for identifying children exposed to maltreatment (e.g., Responding to Child Maltreatment: A Clinical Handbook for Health Professionals, 2022). This is as there is a lack of substantial evidence that early detection through universal screening improves outcomes for children and their families.*

Response to comment 9:

We agree with the reviewer about this important point. We have revised the discussion to emphasise that, consistent with WHO guidance, **there is no substantial evidence that universal screening for child maltreatment improves outcomes for children and families**. We now explain how, in the Dutch context, SCAN&STEPS combines elements of both universal screening and case-finding to enhance awareness and consistency while limiting unnecessary burden and reducing the risk of missed cases. T**his design seeks to address WHO's reservations about universal screening** by combining awareness-raising with targeted case evaluation.

*Revision in text: Universal screening versus case-finding*

The Dutch context combines mandated universal application of SCAN with targeted case evaluation via STEPS for SCAN-positive cases [19]. Intended to reduce the likelihood of missed cases, this hybrid strategy enhances professional awareness and promotes consistency, while safeguarding proportionality by ensuring that further steps are taken only when concerns are identified. It supports physicians in initiating dialogue with parents and reporting to Safe at Home only when necessary to ensure a child's safety. In this way, the universal use of SCAN fosters awareness and clinical enquiry, while avoiding unnecessary burden for families, **thereby addressing key concerns expressed in** line with WHO recommendations [28,29]. Our findings show that compliance improved, more children were screened, and the proportion of positives remained stable at 2.5%, suggesting that SCAN may increase awareness. Whether this translates into improved protection of children remains uncertain. Rigorous evaluation of the full care pathway (including medical follow-up, forensic consultation, voluntary support, and referral to Safe at Home) is needed to determine the predictive value and real-world effectiveness of SCAN&STEPS. The uniform nationwide application of SCAN&STEPS in the Netherlands, offers a unique opportunity to generate insights on performance and outcomes, particularly given the ongoing debate on the effectiveness of universal and targeted approaches [30–32].

Comment 10:

> *The Dutch LECK network is known for applying a Bayesian approach to child maltreatment evaluations (van Rijn et al., BMJ Open, 2019). However, there is insufficient understanding of the positive and negative predictive values of the SCAN instrument, which is critical for assessing its diagnostic utility. Without data on key characteristics and baseline prevalence in the studied population, it is not possible to calculate likelihood ratios, making it difficult to determine how well SCAN differentiates between true and false cases of concern. Given that screening in a low-prevalence population by definition carries a significant risk of false positives, further analysis may be necessary to evaluate the instrument's performance and its implications for clinical decision-making.*

Response to comment 10:

We thank the reviewer for this important observation we and fully agree. As assessing diagnostic accuracy was beyond the scope of this dataset, we focused on demonstrating that SCAN&STEPS implementation can influence professional behaviour. We have added to the discussion that the predictive values and likelihood ratios of SCAN remain unknown and require evaluation in future research, particularly given the risk of false positives in low-prevalence populations. We are preparing further analyses with an appropriate dataset to examine the diagnostic performance and clinical impact of SCAN.

*Revision in text:* Diagnostic accuracy could not be assessed in this study; future research is needed to evaluate SCAN&STEPS' performance.

Comment 11:

> *Case-finding approaches is exemplified in the method used by Lindberg et al. (ref 30, 31 in the manuscript), which selectively identifies a population with a higher baseline prevalence of maltreatment building on evidence-based clinical decision rules and specific presenting conditions (e.g., manifest injuries in children under six years). These distinctions are important in evaluating the feasibility, sensitivity, and real-world applicability of different screening strategies in emergency care.*

*It is crucial to be explicit about the available interventions for children and families identified through screening. When screening for any disease or condition, it is important to ensure that a clear intervention treatment or management pathway is defined for those who screen positive in order to begin to understand whether an intervention ultimately leads to improved outcomes for the child. As understood, Dutch healthcare professionals have an obligation to arrange help for families when there are concerns for child maltreatment.*

*This concern is further compounded by the lack of clarity regarding what happens after a child screens positive—whether the intervention involves referral to Safe at Home, follow up healthcare services, or further interventions in the child protection chain of care. Without clear evidence of fidelity in the decision point physician referral to Safe at Home / retention in healthcare decision tree, or data to otherwise demonstrate improved outcomes, the discussion should acknowledge these critical gaps and frame SCAN's implementation within the broader debate on the effectiveness and risks of universal child maltreatment screening.*

<u>Response to comment 11:</u>

We thank the reviewer for this valuable comment. We have revised the discussion to distinguish universal screening from case-finding (e.g. Lindberg et al.) and clarified the Dutch pathway. We also acknowledge uncertainties regarding the consistency and outcomes of these follow-up steps and have framed SCAN&STEP within the broader international debate on the effectiveness and risks of universal screening in the discussion.

*Revision in text:* This will further enable evaluation across this chain, an essential step towards reducing the impact of child maltreatment. While SCAN&STEPS demonstrates clear potential within the Dutch context, broader international adoption should carefully account for variations in child abuse policies and healthcare systems.

<u>Comment 12:</u>

*These issues are especially important when clinicians—encouraged by the published validation of a tool like SCAN—adapt it to different legislative and policy contexts. For example, SCAN is currently being piloted in Sweden, where robust mandatory reporting laws – to my understanding -- mean that a child who checked positive on CAST screening at an ED triage would automatically trigger a CPS report. Such differences in child protection systems can significantly alter the consequences of screening, reinforcing the need to carefully evaluate predictive values, unintended consequences, and system capacity before broader implementation. The discussion section should include a stronger statement of caution and limitations, acknowledging the lack of evidence linking universal screening to improved outcomes, the absence of predictive validity data, and the risk of false positives in a low-prevalence population. The discussion does not currently consider how SCAN functions in diverse policy environments and systems, which is critical for its adaptation elsewhere. A more explicit discussion of these issues would ensure that policymakers and clinicians interpret the findings with appropriate caution and recognise the need for further validation before wider adoption.*

<u>Response to comment 12:</u>

We thank the reviewer for this valuable comment. The discussion now highlights the lack of outcome and predictive validity data, the risk of false positives, and the need for validation across diverse child protection systems before wider adoption.

*Revision in text:* This will further enable evaluation across this chain, an essential step toward reducing the impact of child maltreatment. While SCAN&STEPS demonstrates clear potential within

the Dutch context, broader international adoption should carefully account for variations in child abuse policies and healthcare systems.
